# Management of Colonic Intramural Hematoma Complicated by Intestinal Obstruction in a Patient With Hemophilia: A Case Report and Literature Review

**DOI:** 10.7759/cureus.111044

**Published:** 2026-06-17

**Authors:** Mohamed Enaimi, Achraf Bahi, Ayoub Bouaiyda, Achraf Jeddab, Mohammed Rabi Andaloussi, Abdelhamid Jaafari, Khalil Mounir, Mustapha Bensghir

**Affiliations:** 1 Department of Anesthesiology and Intensive Care, Mohammed V Military Teaching Hospital, Rabat, MAR; 2 Department of General Surgery, Mohammed V Military Teaching Hospital, Rabat, MAR; 3 Surgical Intensive Care Unit, Mohammed V Military Teaching Hospital, Rabat, MAR

**Keywords:** colonic intramural hematoma, conservative management, factor viii, hemophilia, intestinal obstruction

## Abstract

Colonic intramural hematoma is an exceptional complication of hemophilia. We report the case of an 18-year-old adolescent with severe hemophilia A who presented with a large colonic intramural hematoma complicated by intestinal obstruction following minor abdominal trauma. Conservative management, combining factor VIII replacement therapy, initial resuscitation, and bowel rest, resulted in a favorable outcome, with complete resolution of the hematoma and no need for surgical intervention. This case highlights the effectiveness of early medical management in this rare clinical situation.

## Introduction

Hemophilia is a rare inherited bleeding disorder caused by a deficiency of coagulation factor VIII (hemophilia A) or factor IX (hemophilia B). Worldwide, hemophilia affects approximately 1 in 5,000 male births for hemophilia A and 1 in 25,000-30,000 male births for hemophilia B, with an estimated global prevalence exceeding 800,000 individuals. Patients with severe disease or inhibitors are particularly susceptible to spontaneous or trauma-related bleeding, predominantly involving joints and muscles [1,2]. Gastrointestinal bleeding accounts for only 10-25% of hemorrhagic episodes in hemophilia, while intramural hematomas of the gastrointestinal tract are exceptionally rare, especially when localized to the colon [3]. These lesions may result in bowel obstruction, ischemia, perforation, or abdominal compartment syndrome, posing significant diagnostic and therapeutic challenges [1,3].

We report a case of traumatic colonic intramural hematoma in a patient with hemophilia A that was successfully managed conservatively with factor replacement therapy and supportive care.

## Case presentation

An 18-year-old male patient with severe hemophilia A (baseline factor VIII activity <1%) and no detectable factor VIII inhibitors, diagnosed at the age of 15 years, was admitted to the emergency department following isolated blunt abdominal trauma caused by a stone impact. He presented with severe abdominal pain associated with progressive abdominal distension and cessation of bowel transit, suggestive of acute intestinal obstruction.

On admission, the patient was conscious and neurologically intact. Hemodynamic assessment revealed arterial hypotension associated with tachycardia, while respiratory examination showed tachypnea with preserved oxygen saturation on room air. Abdominal examination demonstrated marked abdominal distension with generalized guarding. Initial biological investigations are summarized in Table 1.

**Table 1:** Initial laboratory findings on admission

Laboratory parameter	Result	Reference values
Hemoglobin	8.2 g/dL	13–17 g/dL
Platelet count	217,000/mm³	150,000–400,000/mm³
Prothrombin time (PT)	57%	70–100%
aPTT ratio	2.2	0.8–1.2
Fibrinogen	2.5 g/L	2–4 g/L
Sodium	131 mmol/L	135–145 mmol/L
Chloride	91 mmol/L	98–107 mmol/L
Potassium	3.73 mmol/L	3.5–5 mmol/L
Bicarbonate reserve	17 mmol/L	22–28 mmol/L
Creatinine	6 mg/L	6–12 mg/L
Urea	0.22 g/L	0.15–0.45 g/L
AST	29 IU/L	<40 IU/L
ALT	18 IU/L	<41 IU/L
Gamma-glutamyl transferase	53 IU/L	10–55 IU/L
Total bilirubin	10 mg/L	3–12 mg/L
Lipase	12 IU/L	13–60 IU/L
Baseline factor VIII activity	<1%	50–150%
Factor VIII inhibitor	<0.6 BU/mL	Negative

Thoracoabdominopelvic computed tomography performed on admission demonstrated a large colonic intramural hematoma measuring 90 × 130 × 18 mm, causing significant luminal narrowing with upstream bowel distension consistent with intestinal obstruction (Figure 1).

**Figure 1:**
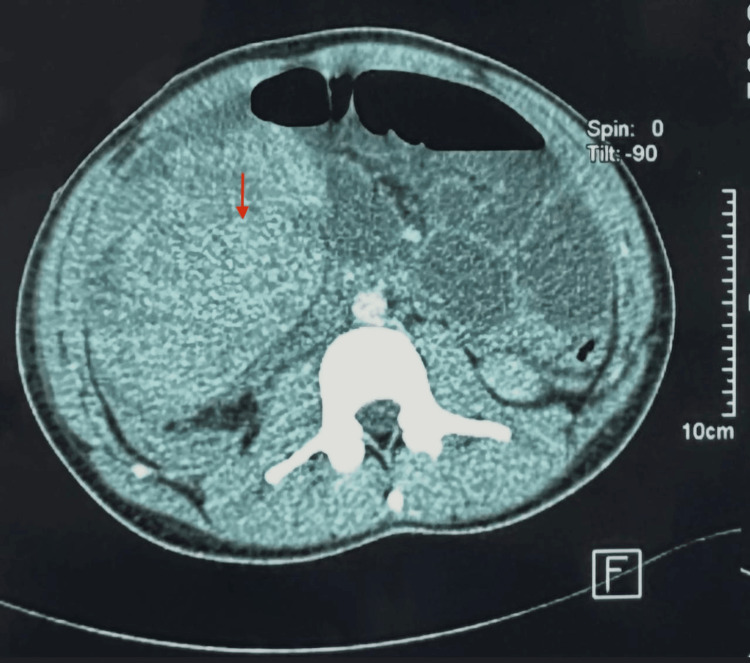
Initial contrast-enhanced axial abdominal computed tomography demonstrating a large colonic intramural hematoma (red arrow) measuring 90 × 130 × 18 mm, causing marked luminal narrowing with upstream intestinal obstruction.

Initial management consisted of hemodynamic resuscitation, including intravenous fluid expansion, transfusion of packed red blood cells, and transient norepinephrine support, which was rapidly discontinued after stabilization. In the absence of indications for emergency surgery, the patient was admitted to the intensive care unit for conservative management without surgical intervention.

Treatment consisted of factor VIII replacement therapy at a dose of 3,200 IU/day for five days, aiming to achieve adequate hemostatic factor VIII levels in accordance with current international recommendations for major bleeding episodes in patients with hemophilia. Strict bowel rest, nasogastric decompression, intravenous fluid resuscitation with electrolyte correction, and parenteral nutrition were also implemented.

Follow-up abdominal computed tomography performed four days after the initiation of conservative treatment demonstrated partial regression and liquefaction of the colonic intramural hematoma, with associated improvement in bowel distension (Figure 2). The clinical course was favorable, with progressive resolution of abdominal pain and recovery of bowel transit without the need for surgical intervention. At the two-month follow-up, the patient remained asymptomatic, and abdominal CT imaging demonstrated near-complete regression of the colonic intramural hematoma, with complete resolution of the bowel obstruction (Figure 3).

**Figure 2:**
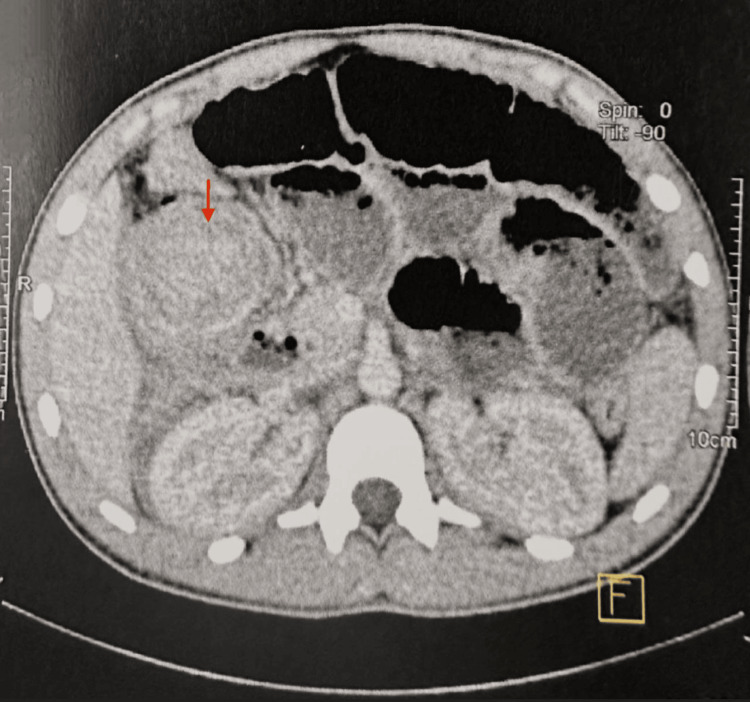
Follow-up abdominal computed tomography performed four days after conservative treatment demonstrating partial regression with liquefaction of the colonic intramural hematoma (red arrow) and improvement of bowel distension.

**Figure 3:**
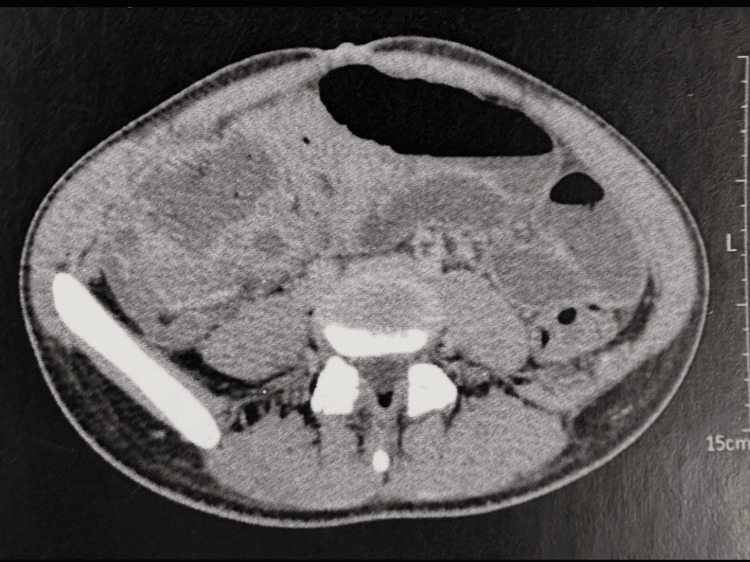
Late follow-up abdominal computed tomography demonstrating near-complete regression of the colonic intramural hematoma after conservative treatment without surgical intervention.

## Discussion

Hemophilia is a rare inherited hemorrhagic disorder caused by a deficiency of coagulation factor VIII (hemophilia A) or factor IX (hemophilia B), resulting from a mutation of the X chromosome. It is transmitted as an X-linked recessive condition, predominantly affecting males, while females are usually asymptomatic carriers [4]. Hemophilia A accounts for approximately 85% of cases, whereas hemophilia B represents about 15%. The incidence is estimated at 1 in 5,000 male births for hemophilia A and 1 in 25,000 for hemophilia B [5]. Clinically, the disease is characterized by a tendency to spontaneous or trauma-induced bleeding, particularly involving muscles, joints, and occasionally internal organs. Severe forms (<1% clotting factor activity) carry the highest risk of life-threatening complications, including gastrointestinal and intracranial hemorrhages [1].

In patients with hemophilia, gastrointestinal bleeding represents a potentially serious complication, although it remains relatively uncommon compared with hemarthroses and muscle hematomas. Digestive hemorrhages may occur spontaneously, particularly in patients with severe disease who are untreated or poorly controlled, or may be triggered by abdominal trauma, endoscopic procedures, surgery, or peptic ulcer disease [1]. The most frequently involved sites are the duodenum and jejunum, which are particularly susceptible to the development of intramural hematomas. Less commonly, the stomach, colon, or rectum may be affected.

When the colon is involved, bleeding may manifest as a colonic obstruction syndrome, characterized by localized abdominal pain, abdominal distension, cessation of flatus and stool passage, and occasionally signs of peritoneal irritation. This presentation is exceptionally rare, especially in a post-traumatic context, but has been reported in the literature in both adult and pediatric populations [7].

The diagnosis of colonic hemorrhage in hemophilic patients relies on a combination of clinical, biological, and radiological findings. Clinically, symptoms vary according to the location and severity of bleeding and may include acute abdominal pain, abdominal distension, vomiting, bowel obstruction, melena or hematemesis, and, in severe cases, signs of hemorrhagic shock. In hemophilic patients, even mild gastrointestinal symptoms should prompt consideration of an intramural intestinal hematoma, particularly in the setting of recent trauma or a history of spontaneous bleeding episodes. Biologically, findings typically include a prolonged activated partial thromboplastin time (aPTT), markedly reduced factor VIII levels in hemophilia A or factor IX levels in hemophilia B, and anemia in cases of active bleeding. Screening for inhibitors should be systematically performed, especially in patients receiving long-term replacement therapy. Imaging plays a pivotal role in diagnosis, with contrast-enhanced abdominal computed tomography (CT) being the modality of choice. CT imaging typically demonstrates concentric bowel wall thickening, often hyperdense, consistent with an intramural hematoma. It may also reveal signs of bowel obstruction, such as proximal bowel dilatation and air-fluid levels, and, in advanced cases, absence of mural enhancement suggestive of ischemia or necrosis [8].

The management of colonic intramural hematomas in patients with hemophilia poses a significant clinical challenge due to the high risk of bleeding and the potential for severe complications related to gastrointestinal involvement. In the absence of alarming signs, treatment is generally conservative, combining factor replacement therapy, bowel rest, and close clinical monitoring. However, surgical intervention may become necessary in the presence of complications such as persistent or worsening obstruction, bowel ischemia, perforation, or failure of conservative management. Therapeutic decision-making therefore depends on several factors, including the severity of hemophilia, the presence of inhibitors, the size and location of the hematoma, and the patient’s clinical course.

In order to identify risk factors associated with the need for surgical intervention in cases of colonic hematoma among hemophilic patients, we reviewed 17 cases reported in the literature (Table 2). Each case was analyzed according to several clinical variables, including age, type of hemophilia, hematoma location, presenting symptoms, type of treatment (conservative or surgical), and clinical outcome. This review provides insight into the clinical characteristics that may be associated with the need for surgical intervention and helps identify situations in which conservative management may be successful.

**Table 2:** Clinical characteristics, management strategies, and outcomes of colonic hematomas in patients with hemophilia: analysis of 17 reported cases and contribution of our case

Age/Sex	Year of publication	Type of hemophilia	Hematoma location	Clinical symptoms	Treatment	Outcome	Reference
18 y / M	2026	Severe Hemophilia A	Colon	Abdominal pain, abdominal guarding, bowel obstruction	Factor VIII	Recovery without complications	Present case
10 y / M	2019	Severe hemophilia A	Rectum	Abdominal pain, tenesmus, persistent constipation	–	–	[4,5]
15 y / M	2015	Hemophilia A	Cecum	Abdominal pain, vomiting, anorexia	Exploratory laparotomy	Recovery without complications	[6,7]
7 y / M	2014	Mild hemophilia A (inhibitor >10 BU/mL)	Sigmoid colon	Rectal bleeding, suprapubic colonic pain	rFVIIa, aPCC	Hematoma resolution	[8,9]
17 y / M	2009	Severe hemophilia A without inhibitors	Sigmoid colon	Abdominal swelling, distension	Factor VIII infusion	Regression of hemoperitoneum	[10]
55 y / M	2008	Hemophilia A	Sigmoid colon	Abdominal pain, distension, compartment syndrome	Emergency laparotomy	Recovery without complications	[9]
29 y / M	2007	Severe hemophilia A	Cecum, ascending and transverse colon	Pain, vomiting, rectal bleeding	Factor VIII infusion	Resolution of intussusceptions	[11]
65 y / M	2006	Hemophilia A (inhibitor: 1 BU/mL)	Cecum	Distension, pain, rectal bleeding	Conservative treatment then laparotomy	Recovery without complications	[12]
26 y / M	1999	Severe hemophilia A	Rectum	Abdominal pain, constipation	–	Symptom resolution	[13]
38 y / M	1990	Moderate hemophilia A	Ascending colon	Pain, palpable mass	Right hemicolectomy	Death (septicemia)	[14]
25 y / M	1987	Hemophilia A with inhibitors	Cecum	Pain, distension, hematemesis	Right hemicolectomy	Recovery without complications	[15]
23 y / M	1985	Hemophilia B	Cecum	Pain, vomiting, anorexia	Surgery	–	[16]
12 y / M	1982	Hemophilia A	Sigmoid colon to rectum	Pain, pallor, peritonitis	Laparotomy	Death	[10]
49 y / M	1979	Hemophilia A	Ascending colon	Appendicitis-like symptoms	Bypass, anastomosis	–	[17]
13.5 y / M	1977	Severe hemophilia A	Colon	Pain, vomiting	Barium enema, cryoprecipitate	Improvement	[18]
9 y / M	1977	Hemophilia A	Descending colon	Pain, melena, severe anemia	Cryoprecipitate	Recovery	[18]
36 y / M	1972	Mild hemophilia A	Sigmoid colon	Constipation, distension, bleeding	Resection, cryoprecipitate	Resolution	[19]
12 y / M	1956	Moderate hemophilia A	Transverse colon	Abdominal pain, rectal bleeding	Cryoprecipitate, strict rest	Improvement	[20]

The analysis of the 17 cases reported in the literature highlighted several clinical features that may influence the course of colonic intramural hematomas in patients with hemophilia. Although the limited number of reported cases precludes definitive conclusions, certain characteristics appeared to be associated with a higher risk of complications and the need for surgical intervention. Conversely, early diagnosis, prompt factor replacement therapy, and the absence of severe complications were commonly observed in patients successfully managed conservatively. In our case, these factors may have contributed to the favorable outcome.

Symptom resolution with conservative management has been reported in several cases, demonstrating that infusion of coagulation factors, particularly factor VIII, cryoprecipitate, activated factor VII, or activated prothrombin complex concentrate, may be sufficient to treat intramural hematomas and improve their clinical course [9]. The absence of signs of severe complications, such as when abdominal pain or constipation is not associated with peritonitis, significant abdominal distension, or infection, also supports a conservative therapeutic approach. Patients without inhibitors or with less severe forms of hemophilia appear to respond more favorably to medical treatment. Published cases show that in moderate forms or in patients without inhibitors, factor replacement therapy often leads to a favorable outcome when symptoms remain mild, and the hematoma tends to regress under substitution therapy. The absence of intestinal perforation or true mechanical obstruction on imaging represents another major argument in favor of conservative management; when imaging studies do not reveal perforation, marked obstruction, or other severe complications, administration of coagulation factors and symptomatic treatment is often sufficient [9-11,17]. In our case, the absence of peritoneal signs, early clinical improvement, and radiological regression of the hematoma further supported the indication for strict conservative treatment.

First-line management of intramural hematomas in patients with hemophilia is based on rapid replacement of factor VIII to control bleeding and prevent extension of the hematoma. The therapeutic goal is to achieve a factor level of at least 50% in moderate forms and at least 80% in severe forms [4,5]. Replacement therapy may be administered either as repeated boluses every 8 to 12 hours or as a continuous infusion of 50 IU/kg/day in cases of significant bleeding. Close biological monitoring is essential and should include measurement of factor VIII levels as well as systematic screening for inhibitors that may limit treatment efficacy [17]. In our case, the rapid clinical improvement and early radiological regression confirmed the effectiveness of the administered replacement therapy.

A colonic intramural hematoma may cause mechanical obstruction of the intestinal lumen. In most published cases, conservative management is preferred to allow gradual resorption of the hematoma. Management includes placement of a nasogastric tube with intermittent suction to reduce vomiting and abdominal distension, intravenous fluid resuscitation with correction of electrolyte disturbances, and strict bowel rest, with the addition of parenteral nutrition in cases of prolonged obstruction. Daily clinical monitoring is essential to assess pain, bowel function, and the possible emergence of complications [6,10]. In our case, resolution of the obstruction by the fourth day illustrates the effectiveness of conservative treatment when initiated early.

Severe hemophilia A is frequently involved in complicated forms requiring invasive management. Patients with inhibitors, particularly those with hemophilia A and inhibitor titers exceeding 10 Bethesda units, have a significantly increased risk of abdominal complications and a more limited response to replacement therapy [5,17]. Younger age, especially in children and adolescents, also appears to be associated with a higher probability of abdominal complications [2,8]. The location of the hematoma further influences disease severity; involvement of the cecum, sigmoid colon, or rectum is often associated with severe pain, abdominal distension, or rectal bleeding, and with a higher risk of unfavorable evolution [10,11]. The presence of severe symptoms such as marked distension, intense pain, or abdominal compartment syndrome represents another major indicator of complication [8,17]. Comorbidities, particularly peritonitis, which may result from secondary intestinal perforation caused by the hematoma, further increase the risk of surgery and severe complications [10,16]. In our case, although the patient was an adolescent, rapid hemodynamic stabilization, early factor VIII replacement therapy, and the absence of severe complications likely contributed to the favorable outcome.

Surgical intervention in hemophilic patients with colonic intramural hematoma remains uncommon but becomes necessary when conservative management fails or when signs of severe complications develop. The main indications reported in the literature include failure of replacement therapy, defined as the absence of clinical or radiological improvement despite repeated administration of coagulation factors, as well as major complications such as peritonitis, intestinal ischemia, perforation, abdominal compartment syndrome, or persistent hemorrhage leading to hemodynamic instability [10,11,16]. Progressive abdominal distension, intractable vomiting, significant rectal bleeding, or the appearance of an abdominal mass suggestive of complete obstruction may also prompt surgical intervention. In several published cases, surgery was required to manage severe obstruction or progressive abdominal infection, sometimes involving hemicolectomy or segmental resection [6,17]. In our case, none of these indications were present, as the patient’s clinical stability, absence of peritonitis, and early regression of the hematoma on CT imaging allowed continuation of conservative treatment without surgery.

Radiological evolution of intramural hematomas is generally favorable under medical management, with progressive resorption observed within one to three weeks. Abdominal CT performed on the third and seventh days is commonly used to assess hematoma size reduction and to detect potential complications [11]. In our observation, radiological follow-up at day 3 showed early liquefaction, confirming the effectiveness of the therapeutic approach. Restoration of bowel transit by day 4 is consistent with timelines reported in the literature.

In patients with hemophilia, prevention of gastrointestinal bleeding relies on regular hematological follow-up to optimize prophylactic factor VIII therapy. Patient education regarding the risks of abdominal trauma and the warning signs of internal bleeding is essential. Early management of digestive complications also plays a key role in preventing recurrence [12].

A simplified diagnostic and therapeutic approach based on the literature review and our clinical observation is proposed in Figure 4.

**Figure 4:**
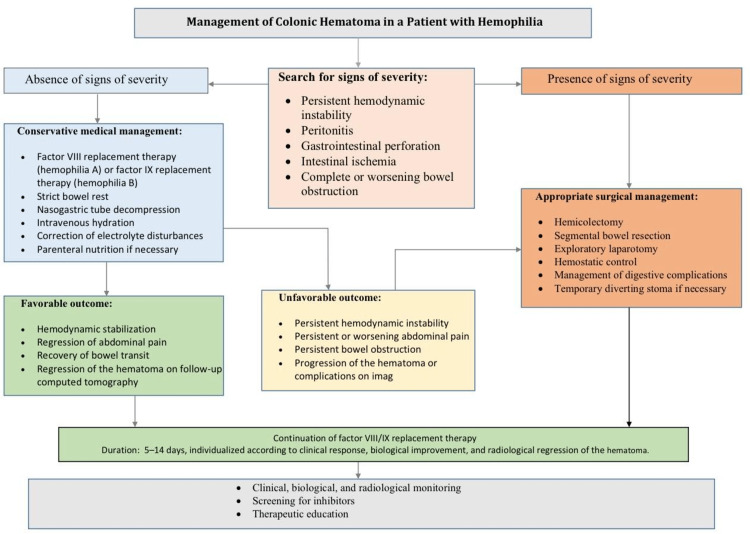
Simplified management algorithm of colonic hematoma in patients with hemophilia.

## Conclusions

Colonic intramural hematoma is a rare but important gastrointestinal complication in patients with hemophilia. It should be considered in hemophilic patients presenting with abdominal pain and bowel obstruction. Contrast-enhanced CT plays a key role in diagnosis. Early conservative management with coagulation factor replacement, bowel rest, and supportive care can lead to favorable outcomes and may avoid unnecessary surgery. Surgical intervention should be reserved for complicated cases or failure of medical treatment.
